# Natural Language Processing Algorithms for Normalizing Expressions of Synonymous Symptoms in Traditional Chinese Medicine

**DOI:** 10.1155/2021/6676607

**Published:** 2021-10-11

**Authors:** Lu Zhou, Shuangqiao Liu, Caiyan Li, Yuemeng Sun, Yizhuo Zhang, Yuda Li, Huimin Yuan, Yan Sun, Fengqin Xu, Yuhang Li

**Affiliations:** ^1^Beijing University of Chinese Medicine, School of Traditional Chinese Medicine, Beijing 100029, China; ^2^Beijing University of Chinese Medicine, School of Traditional Chinese Medicine, TCM Information Science Research Center, Beijing 100029, China; ^3^Xiyuan Hospital of China Academy of Chinese Medical Sciences, Beijing 100091, China

## Abstract

**Background:**

The modernization of traditional Chinese medicine (TCM) demands systematic data mining using medical records. However, this process is hindered by the fact that many TCM symptoms have the same meaning but different literal expressions (i.e., TCM synonymous symptoms). This problem can be solved by using natural language processing algorithms to construct a high-quality TCM symptom normalization model for normalizing TCM synonymous symptoms to unified literal expressions.

**Methods:**

Four types of TCM symptom normalization models, based on natural language processing, were constructed to find a high-quality one: (1) a text sequence generation model based on a bidirectional long short-term memory (Bi-LSTM) neural network with an encoder-decoder structure; (2) a text classification model based on a Bi-LSTM neural network and sigmoid function; (3) a text sequence generation model based on bidirectional encoder representation from transformers (BERT) with sequence-to-sequence training method of unified language model (BERT-UniLM); (4) a text classification model based on BERT and sigmoid function (BERT-Classification). The performance of the models was compared using four metrics: accuracy, recall, precision, and F1-score.

**Results:**

The BERT-Classification model outperformed the models based on Bi-LSTM and BERT-UniLM with respect to the four metrics.

**Conclusions:**

The BERT-Classification model has superior performance in normalizing expressions of TCM synonymous symptoms.

## 1. Introduction

Traditional Chinese medicine (TCM) symptoms are recorded by TCM practitioners who sometimes use different words when recording the same symptoms, as a consequence of their diverse experience and educational background. These variations in words lead to the phenomenon known as “one symptom with different literal expressions,” which is prevalent in TCM medical records. Wang et al. [1] reported that approximately 80% of TCM symptoms were recorded with multiple expressions. Although the literal expressions of these symptoms are different, they have the same meaning, and their use does not affect understanding. Thus, the use of these alternative symptoms does not affect the pathogenesis diagnosis. In summary, TCM symptoms that have the same meaning but different literal descriptions are known as TCM synonymous symptoms. For example, the symptom “lack of appetite” (纳减) can also be expressed as “loss of appetite” (纳差) or “decreased appetite” (食欲减低). They all mean a reduced desire to eat and are used in the description of spleen Qi deficiency (脾气虚).

It is essential to explore and analyze TCM medical records for the purpose of TCM modernization [2, 3]. However, the abundance of synonymous symptoms in TCM medical records hinders systematic scientific knowledge discovery. Referring to the TCM terminology [4] published by relevant authorities, it is possible to establish a TCM thesaurus and then normalize each symptom in TCM medical records to a symptom that has the same meaning in the thesaurus, so that TCM synonymous symptoms would have uniform literal expressions. That is, TCM symptom normalization is a feasible method for handling TCM synonymous symptoms. However, manual TCM symptom normalization is time-consuming and labor-intensive because of the large and growing quantity of TCM electronic medical records.

Natural language processing (NLP), which has experienced extraordinary development in recent years, provides valuable support for the automatic processing of text data, such as language translation [5], question answering [6], and information processing of medical texts [7–10]. This success suggests that the NLP technology will be effective for normalizing the expression of TCM synonymous symptoms.

In previous work, researchers have proposed some NLP-based normalization models for biomedical fields, such as Word2Vec [11], Jaccard similarity [12], DNorm [13], and BERT-based ranking [14] from the perspective of similarity matching. In addition, from the perspective of named entity recognition (NER), there are transition-based [15] models and Bi-LSTM-CNNs-CRF [16]. Although the performance of these models is satisfactory according to the published reports, there are two problems that are worthy of further exploration, from the perspectives of their applicability to normalizing TCM symptoms and the modeling concepts of the NLP model:

1. With regard to applicability, the above models are used for normalizing multiple synonymous terms to one term. However, they are not suitable for cases in which synonymous symptoms correspond to multiple normalized symptoms. For example, “less white sputum and difficult to expectorate” (痰少色白难咳) and “less white phlegm and not easy to expectorate” (少量白痰且不易咳出) are synonymous symptoms, should be normalized to “less phlegm” (痰少), “white phlegm” (痰白), and “expectoration difficulties” (痰难咳出).
2. With regard to the modeling concept, approaches from the perspectives of similarity matching and NER have been reported. However, many models constructed from the perspectives of sequence generation and text classification have also shown excellent performance and applicability in NLP tasks [17, 18]. Therefore, it is necessary to explore the applicability of sequence generation and text classification to this normalization task and investigate whether better performance can be achieved.

According to the above statement, the objective of this study is to develop normalization models for normalizing the expressions of TCM synonymous symptoms from the perspective of sequence generation and text classification and to compare and analyze the applicability and performance of the models, so as to select the best model.

## 2. Methods

**Figure 1:**
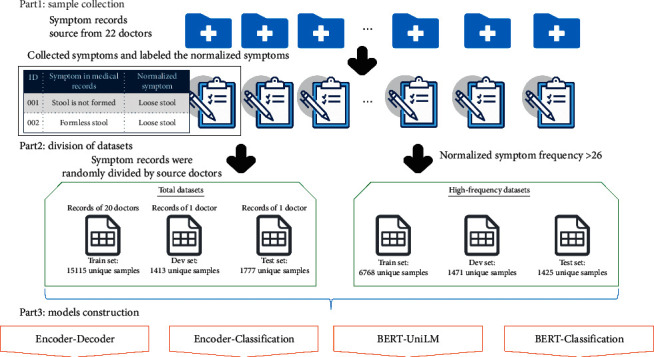
Workflow of the research process.

The workflow of this study is shown in Figure 1. It can be divided into three parts: (1) collecting TCM symptoms from medical records (referred to as sample collection), (2) preparing training, development, and test data sets (referred to as division of data sets), and (3) constructing models for normalizing expressions of TCM synonymous symptoms (referred to as model construction).

### 2.1. Data Sources and Labeling

In total, 3,252 medical records, recorded by 22 TCM doctors on the platform of the “Heritage Program of Chinese Well-Known Experts” [19], were collected. The symptoms in the medical records were regarded as the original symptoms, each of which was then labeled by the corresponding normalized symptom, according to the *TCM Thesaurus* (from the Beijing University of Chinese Medicine TCM Information Science Research Center). Two researchers, who had obtained the qualification of TCM practicing physician and been trained by the provider of the *TCM Thesaurus*, performed the labeling work. Two additional experts in the *TCM Thesaurus* checked the labeling results independently, and inconsistent labeling results were submitted to a third expert for review and discussion to ensure consistency.

There are two forms of original symptoms in medical records: single symptoms and complex symptoms. A single symptom is an original symptom that corresponds to only one clinical manifestation; such a symptom was labeled as one normalized symptom by referring to the *TCM Thesaurus*. For example, “thinning and shapeless stool” was labeled as “loose stool.” A complex symptom is an original symptom that corresponds to multiple clinical manifestations; such a symptom was labeled as multiple normalized symptoms. For example, “dry and itchy throat” was labeled as “dry throat” and “itchy throat” by referring to the *TCM Thesaurus*.

In total, 16,808 nonrepetitive original symptoms were collected from the 3,252 medical records, corresponding to 1,501 normalized symptoms, of which 339 appeared only once. The collected original symptoms and labeled normalized symptoms served as the input and output data, respectively, of TCM symptom normalization models.

### 2.2. Partition of Data Sets

Two strategies were used to divide the collected data into training, development, and test data sets. The first strategy was to divide the medical records by source doctors randomly. The nonrepetitive original symptoms recorded by one randomly selected doctor, and the corresponding normalized symptoms were used as a development set to set the parameters of the model. The nonrepetitive original symptoms recorded by another randomly selected doctor, and the corresponding normalized symptoms were used as a test set to observe the ability of the model to normalize the expression of TCM symptoms. The nonrepetitive original symptoms recorded by the 20 other doctors, and the corresponding normalized symptoms were used as the training set. These data sets were called total data sets (TDS). This data set division is suitable for evaluating the performance of the TCM symptom normalization models in practical applications.

**Figure 2:**
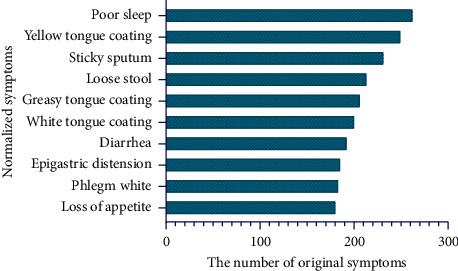
The ten most frequent normalized symptoms in the collected medical records.

The second strategy for dividing the collected data into training, development, and test data sets was based on high-frequency normalized symptoms. These data sets were called high-frequency data sets (HFDS). According to Zipf's law [20], $N=-1+\sqrt{1+8\times I_{1}}/2$ (*N* is the threshold between high-frequency and low-frequency, and *I*_1_ is the number of normalized symptoms that only appeared once). Normalized symptoms with a frequency greater than 26 were defined as high-frequency normalized symptoms. The high-frequency normalized symptoms and the corresponding original symptoms were included in the HFDS. The ten most frequent normalized symptoms and their corresponding numbers of original symptoms are shown in Figure 2. In the HFDS, 70% of the data (6,768 original symptoms and the corresponding normalized symptoms) were randomly selected as a training set, 15% (1,471 original symptoms and the corresponding normalized symptoms) were as a development set, and 15% (1,425 original symptoms and the corresponding normalized symptoms) were as a test set. The numbers of samples in HFDS and TDS are shown in Table 1.

### 2.3. Model Construction

**Figure 3:**
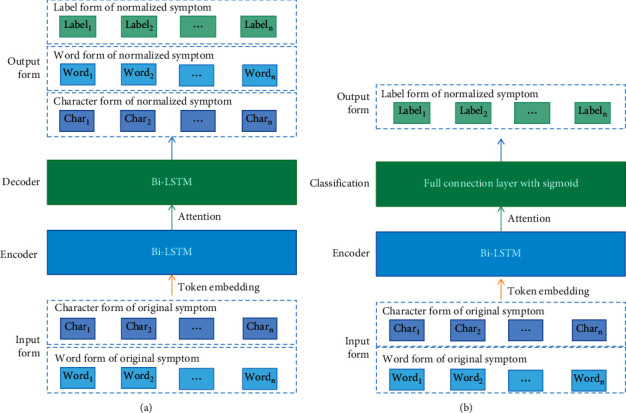
Examples of the (a) Encoder-Decoder and (b) Encoder-Classification models.

From the perspective of text sequence generation, the bidirectional long short-term memory (Bi-LSTM) recurrent neural network (RNN) with the encoder-decoder structure [21], combined with the Luong attention mechanism [22], was used to establish four models for TCM symptom normalization. (1) Encoder (Char)-Decoder (Char) model: the input of the original symptom and the output of the normalized symptom were in character form (multiple output normalized symptoms were separated by “,”). (2) Encoder (Word)-Decoder (Word) model: the input of the original symptom and the output of the normalized symptom were in word form. (3) Encoder (Char)-Decoder (Label) model: the input of the original symptom was in character form, and the output of the normalized symptom was in label form. (4) Encoder (Word)-Decoder (Label) model: the input of the original symptom was in word form, and the output of the normalized symptom was in label form. The structure of the four models was consistent; only the input and output forms were different, as shown in Figure 3(a).

This study also applied the Bi-LSTM and a full connection layer with sigmoid function to explore the feasibility of TCM symptom normalization from the perspective of text classification. In this case, the model output was in label form, and the input was in character or word form (see Figure 3(b)). In the Encoder (Char)-Classification model, the input was in character form; in the Encoder (Word)-Classification model, the input was in word form. The words that were input to the model were obtained from the original symptoms by a segmentation tool [23].

**Figure 4:**
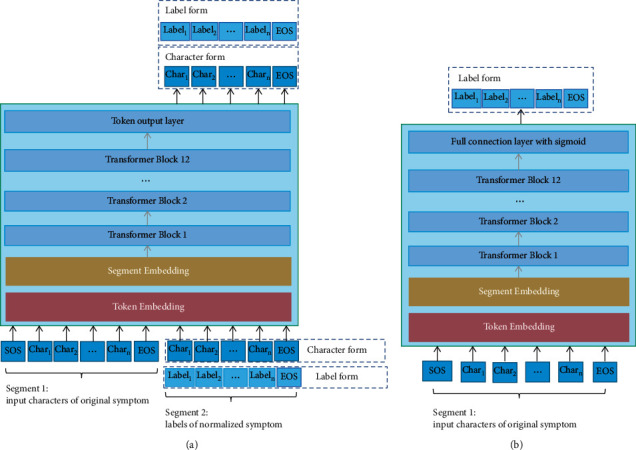
Examples of the BERT-UniLM and BERT-Classification models. (a) This model structure is consistent with BERT. There are 12 transformer blocks. According to the embedding composition of BERT, including segment embedding of segments 1 and 2 and character embedding of original symptom. The token output layer of the model outputs the normalized symptom in character form or label form through a fully connected layer with a softmax function. SOS is the symbol at the start of the sequence, and EOS is the symbol at the end of the sequence. This model was trained by the sequence-to-sequence method of UniLM. (b) This normalization model structure is also based on BERT. In contrast to (a), a full connection layer with the sigmoid function is used as the output layer.

Chinese language pretraining weights, trained on a large number of Chinese corpora, can help achieve better results. Therefore, this study further used the unified language model (UniLM) based on the Chinese pretraining weights of bidirectional encoder representation from transformers (BERT) [18, 24] to construct the TCM symptom normalization model. The training process included first loading the Chinese pretraining weights of BERT (https://storage.googleapis.com/bert_models/2018_11_03/chinese_L-12_H-768_A-12.zip) and then training with the sequence-to-sequence method of UniLM [18]. This training method was based on text sequence generation. Two output forms were used in training: a character-based output form, namely the BERT-UniLM (Char) model, and a label-based output form, namely the BERT-UniLM (Label) model, as shown in Figure 4(a). BERT and a full connection layer with sigmoid function were also used to construct the TCM symptom normalization model, namely the BERT-Classification model, as shown in Figure 4(b). Because the input of the pretraining weights of BERT was in character form, the input of the BERT-based models was also in character form.

### 2.4. Model Parameters

The encoder-decoder models had initialization weights sampled from a random uniform distribution in the range of −0.05–0.05, the dimension of embedding was 300, and the training batch size was 256. Adam was the optimizer [25]. According to the F1-score of the encoder-decoder models on the development set, the best parameter combinations were selected for learning rate (selected from 0.0001, 0.0003, and 0.0005), dropout rate (selected from 0.3 and 0.5), and the number of memory cells (selected from 128, 256, and 512).

For the encoder-classification models, the training batch size was 256. According to the F1-score of the models on the development set, the best parameter combinations were selected for learning rate (selected from 0.005, 0.01, and 0.03), dropout rate (selected from 0.3 and 0.5), and the number of memory cells (selected from 128, 256, and 512).

For the BERT-UniLM and BERT-Classification models, the training batch size was 16, the optimizer was Adam [25], and the learning rate was 0.0003. The other parameters were the default settings of the BERT neural network [24].

The TensorFlow neural network framework (http://www.tensorflow.org/), developed by Google, was used to implement the above models and was combined with NVIDIA GeForce RTX 2080 (11 GB memory) to train the models. When the F1-score of the models in the development set had not improved for 20 epochs, the training was terminated. Even if a fixed random seed number was used, the results from different computers were still biased. Therefore, after setting the model parameters, the modeling process was repeated 10 times; the model performance was evaluated by four metrics and expressed as mean ± standard deviation (SD). The four metrics used were accuracy, precision, recall, and F1-score.  Accuracy=*P*/*T*; Precision=TP/TP+FP ; Recall=TP/TP+FN; and *F*1 − score=2 × Precision × Recall/Precision+Recall. Here, *P* (the correct normalized symptoms of model prediction) is the number of all correct results output by the model, and *T* (total correct normalized symptoms corresponding to the test set) is the number of all tests. TP (true positive) is the number of results produced by the model that were consistent with the actual results, FN (false negative) is the number of correct results that the model failed to output, and FP (false positive) is the number of results produced by the model that were incorrect. The key model parameters and development set results are shown in Tables 2 and 3.

### 2.5. Statistical Analysis

IBM SPSS 20.0 was used to analyze the results. When analyzing the indexes of each group, if the variance between groups was homogeneous and normal distribution was satisfied, one-way ANOVA was used. If the variance was not homogeneous or there was non-normal distribution among groups, the Kruskal–Wallis test was used.

## 3. Results

### 3.1. Performance of Models on Test Data Sets

Generally, the performance of models was better on the HFDS test data set than on TDS. With regard to the model structure, the BERT-UniLM models had more advantages than the Encoder-Decoder models, as shown in Tables 4 and 5. In addition, comparing the BERT-UniLM models with the BERT-Classification model, the BERT-Classification model had more advantages. That is, the BERT-Classification model was the best model for normalizing expressions of TCM synonymous symptoms in this study, on both the HFDS and TDS test data sets.

**Figure 5:**
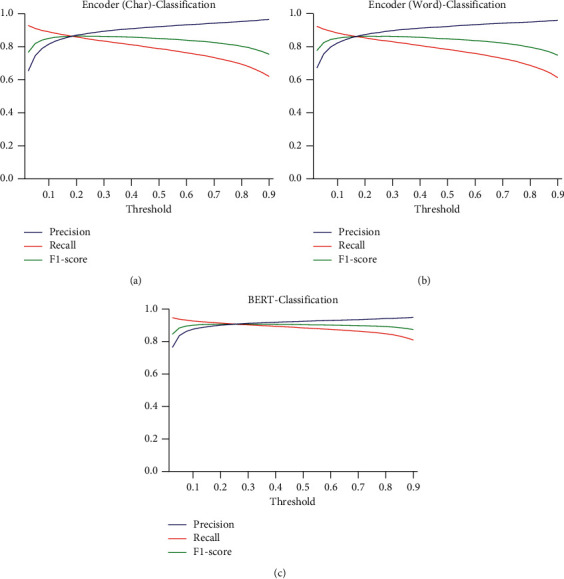
Effects of different thresholds on HFDS. The mean values of precision, recall, and F1-score of the (a) Encoder (Char)-Classification model, (b) Encoder (Word)-Classification model, and (c) BERT-Classification model at different sigmoid output thresholds.

**Figure 6:**
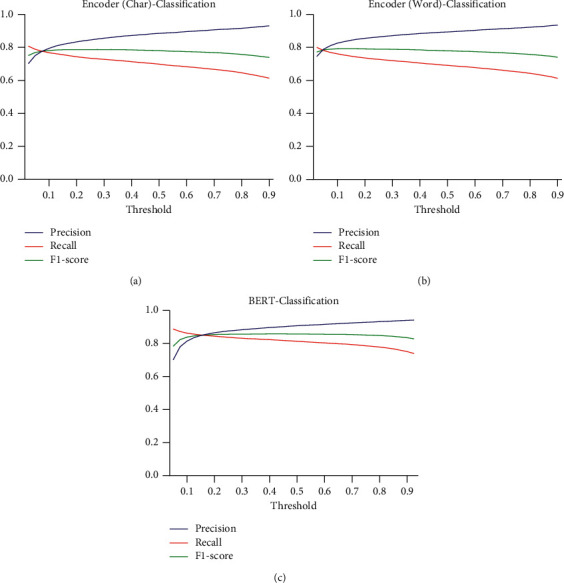
Effects of different thresholds on TDS. The mean values of precision, recall, and F1-score of the (a) Encoder (Char)-Classification model, (b) Encoder (Word)-Classification model, and (c) BERT-Classification model at different sigmoid output thresholds.

The performance of three classification models with different threshold values on HFDS and TDS was explored. With regard to HFDS, when the threshold value was 0.2, the performance of both BERT-Classification and Encoder-Classification was generally the best, as shown in Figure 5. With regard to TDS, the best threshold value was 0.1, as shown in Figure 6. When comparing the BERT-Classification model with the Encoder-Classification models, the BERT-Classification model achieved better results. The accuracy and F1-score were 0.9051 and 0.9073 on the HFDS and 0.8568 and 0.8574 on the TDS, respectively.

The classification-based models have the ability to adjust the output threshold to change the recall. We believe this capability can be used for the retrieval of normalized symptoms. Because retrieval focuses on higher recall, namely, focuses on the outputs contain the correct normalized symptoms. By lowering the output threshold, the models can output the top 5 and 10 normalized symptoms above the threshold. Therefore, the retrieval ability was evaluated by the top 5 and 10 recall, and the results are shown in Table 6.

### 3.2. Performance of Models in Normalizing Single and Complex Symptoms

**Figure 7:**
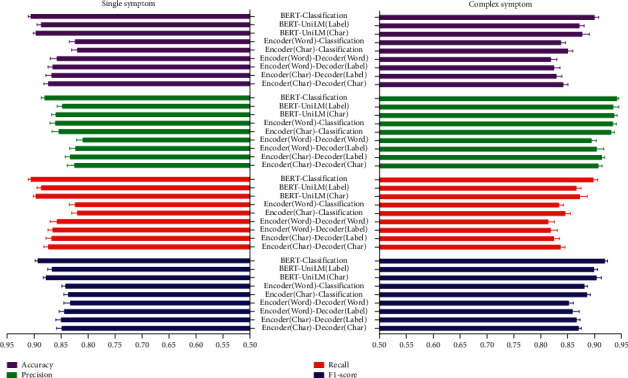
Normalization performance of single and complex symptoms on HFDS.

**Figure 8:**
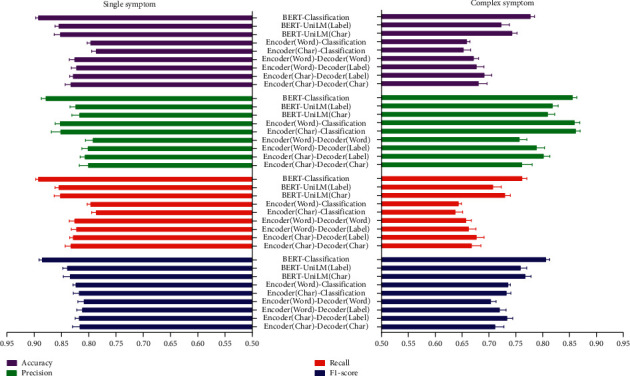
Normalization performance of single and complex symptoms on TDS.

In evaluating the various models for normalizing single symptoms (the original symptoms corresponding to one normalized symptom) and complex symptoms (the original symptoms corresponding to multiple normalized symptoms), we found that the performance of the BERT-Classification model was comprehensively superior, not only on HFDS but also on TDS, as shown in Figures 7 and 8.

### 3.3. Comparison with Other Normalization Models

We also compared the BERT-Classification model with several other models that perform well for normalization, including the state-of-the-art models reported by other researchers. These methods are the Jaccard similarity algorithm [12], Word2Vec with cosine [11], DNorm [13], the transition-based model [15], RNN-CNNs-CRF [16], and BERT-based ranking [14]. The above models were not designed for the normalization of complex symptoms. Therefore, we only compared the performance of models to handle single symptoms (4,555 single symptoms) taken from the HFDS. The 4,555 single symptoms, and their corresponding normalized symptoms, were divided into a training set (70%), a development set (15%), and a test set (15%). The development set was used to select the parameters of each model, except the Jaccard method, for which there is no need to select parameters. The test results showed that the BERT-Classification model performed better than the other methods, as shown in Table 7.

We note that Jaccard similarity, Word2Vec with cosine, DNorm, and BERT-based ranking can output the score of each normalized symptom. Therefore, the models can output the top 5 and 10 normalized symptoms by score ranking to achieve retrieval. We used recall to observe the ability of retrieval, as shown in Table 8. The results show that the BERT-Classification model has advantages in retrieval.

To further demonstrate the advantages of our model, we summarized the test results on HFDS. According to the results, we comprehensively compared the performance and applicability of our model with that of existing models, as shown in Table 9.

## 4. Discussion

The normalization of expressions of TCM synonymous symptoms plays an important role in the collation of medical records, statistical mining, construction of TCM knowledge databases, and construction of TCM medical assistant decision-making systems [9]. The application of NLP technology improves the efficiency of normalization processing. NLP algorithms based on neural networks have been applied in normalizing biomedical texts [13, 14] but not in normalizing the expressions of TCM synonymous symptoms. In this study, multiple models were constructed with NLP algorithms based on Bi-LSTM and the BERT neural network to explore the normalization of expressions of TCM synonymous symptoms.

In TCM synonymous symptom normalization, the performance of normalization and the ability to handle one symptom corresponding to multiple normalized symptoms are crucial to the normalization model. The test results show that our BERT-Classification model outperforms previous models and has the ability as mentioned above, while previous models do not have. In addition, the model also supports retrieve normalized candidate symptoms. Our model can retrieve other candidate normalization symptoms according to original symptoms when the model does not provide suitably normalized symptoms.

These advantages of the model provide technical support for the efficient normalization of TCM synonymous symptoms and make the model highly adaptable in medical situations.

In this study, the accuracy, recall, precision, and F1-score metrics were used to evaluate the performance of each model. The results show that the BERT-Classification model outperformed other existing models with respect to various metrics; these models include the proposed Encoder-Decoder, Encoder-Classification, and BERT-UniLM designed in this study. This is because the performance of NLP models based on neural networks is strongly related to the extracted semantic features, and BERT excels in extracting semantic features [24]. Therefore, the BERT-Classification model, which extracts semantic features using BERT, is advantageous for normalization tasks. BERT-Classification, BERT-UniLM, and BERT-based ranking are all based on the BERT neural network; they differ only in their output layers due to their different modeling concepts. The results suggest that BERT-Classification performs best; therefore, the classification-based modeling concept may be the most conducive to normalizing TCM symptoms.

With regard to applicability, our proposed BERT-Classification model supports both the processing of the original symptoms that correspond to multiple normalized symptoms and the retrieval of normalized symptoms. We use sigmoid as an output function to handle the situation in which each original symptom corresponds to multiple normalized symptoms; this method is effective and outperforms sequence generation methods. Moreover, for the model to support the retrieval of normalized symptoms, it requires a higher recall. Our BERT-Classification model can increase the recall by reducing the output threshold of the sigmoid function and thereby support retrieval.

In contrast to BERT-Classification, the other reported models cannot support both of the above applications simultaneously. Jaccard similarity, DNorm, Word2vec with cosine, and BERT-based ranking pair an original symptom with each normalized symptom and rank the normalized symptoms by their pairing score. Although these models can output multiple normalized symptoms by ranking them for retrieval, when multiple normalized symptoms corresponding to the original symptoms need to be output precisely, it is difficult to decide whether the results (except for the normalized symptom with the highest score) should be output. The Bi-LSTM-CNNs-CRF model is only designed for outputting a single normalized symptom. In addition, because the model is based on the NER modeling concept, it cannot produce multiple candidate normalized symptoms, as the above models can, and therefore cannot be applied to the retrieval task. Although the Encoder-Decoder and BERT-UniLM models support the output of multiple normalized symptoms, they suffer from the same limitations as Bi-LSTM-CNNs-CRF and are not suitable for the retrieval of normalized symptoms.

The HFDS contained only high-frequency samples for modeling and testing, reflecting the performance of the BERT-Classification model under ideal conditions. Conversely, the TDS included both high-frequency and low-frequency samples, reflecting the performance of the model in practical applications. Comparing the results of the model on the two data sets, the performance on TDS was lower than that on HFDS. This suggests that the performance of the model can be improved by increasing the number of low-frequency samples.

## 5. Conclusions

This study constructed models to normalize TCM synonymous symptoms from the perspectives of text classification and sequence generation of NLP. The optimal model is the BERT-Classification model, which outperforms existing reported models in dealing with original symptoms that correspond to a single normalized symptom. Moreover, it also supports original symptoms that correspond to multiple normalized symptoms, and it has the ability to retrieve normalized symptoms. The limitation of this study is that the normalization models only explore symptoms. Whether the models can be used for normalizing other synonymous terms, such as TCM treatment terms and TCM disease terms, remains to be further studied. In addition, the pretrained BERT model based on large-scale corpora plays an important role in improving the model performance; the BERT model trained on corpora from professional medical fields is likely to achieve better results for normalization of medical terms. Therefore, the use of a large number of TCM literature corpora to construct the pretrained model, to improve the normalization performance, also needs further research.

## Figures and Tables

**Table 1:** Numbers of samples in HFDS and TDS.

Data set	HFDS	TDS
Training	6,768	15,115
Development	1,471	1,413
Test	1,425	1,777
Total	9,664	18,305

**Table 2:** Model parameters and development set results on HFDS.

Model	LR	DR	MC	Accuracy	Precision	Recall	F1-score
Encoder (Char)-Decoder (Char)	0.0003	0.5	512	0.8631 ± 0.0042	0.8637 ± 0.0091	0.8587 ± 0.0038	0.8611 ± 0.0053
Encoder (Char)-Decoder (Label)	0.0005	0.5	256	0.8688 ± 0.0046	0.8812 ± 0.0070	0.8623 ± 0.0044	0.8716 ± 0.0048
Encoder (Word)-Decoder (Label)	0.0005	0.3	512	0.8631 ± 0.0042	0.8637 ± 0.0091	0.8587 ± 0.0038	0.8611 ± 0.0053
Encoder (Word)-Decoder (Word)	0.0005	0.3	512	0.8549 ± 0.0055	0.8596 ± 0.0047	0.8468 ± 0.0065	0.8531 ± 0.0052
Encoder (Char)-Classification	0.005	0.3	512	0.8377 ± 0.0060	0.9020 ± 0.0109	0.8414 ± 0.0062	0.8706 ± 0.0058
Encoder (Word)-Classification	0.005	0.5	512	0.8326 ± 0.0061	0.8978 ± 0.0068	0.8335 ± 0.0056	0.8645 ± 0.0043
BERT-UniLM (Char)	0.00003	0.1	N/A	0.8966 ± 0.0027	0.9013 ± 0.0064	0.8920 ± 0.0041	0.8966 ± 0.0025
BERT-UniLM (Label)	0.00003	0.1	N/A	0.8957 ± 0.0042	0.8996 ± 0.0063	0.8895 ± 0.0038	0.8945 ± 0.0039
BERT-Classification	0.00003	0.1	N/A	0.9087 ± 0.0029	0.9216 ± 0.0027	0.9084 ± 0.0034	0.9150 ± 0.0018

*Note.* LR: learning rate; DR: dropout rate; MC: number of memory cells of RNN; N/A: not applicable.

**Table 3:** Model parameters and development set results on TDS.

Model	LR	DR	MC	Accuracy	Precision	Recall	F1-score
Encoder (Char)-Decoder (Char)	0.0005	0.3	512	0.8212 ± 0.0038	0.8307 ± 0.0107	0.8011 ± 0.0044	0.8156 ± 0.0067
Encoder (Char)-Decoder (Label)	0.0003	0.5	512	0.8160 ± 0.0026	0.8379 ± 0.0060	0.7959 ± 0.0030	0.8164 ± 0.0033
Encoder (Word)-Decoder (Label)	0.0003	0.5	512	0.8091 ± 0.0045	0.8320 ± 0.0048	0.7875 ± 0.0052	0.8091 ± 0.0040
Encoder (Word)-Decoder (Word)	0.0001	0.3	256	0.8053 ± 0.0028	0.8167 ± 0.0076	0.7838 ± 0.0034	0.7999 ± 0.0049
Encoder (Char)-Classification	0.01	0.5	512	0.7681 ± 0.0058	0.8876 ± 0.0114	0.7503 ± 0.0051	0.8132 ± 0.0061
Encoder (Word)-Classification	0.01	0.3	512	0.7790 ± 0.0051	0.8913 ± 0.0074	0.7595 ± 0.0060	0.8201 ± 0.0034
BERT-UniLM (Char)	0.00003	0.1	N/A	0.8338 ± 0.0027	0.8399 ± 0.0047	0.8180 ± 0.0034	0.8288 ± 0.0033
BERT-UniLM (Label)	0.00003	0.1	N/A	0.8219 ± 0.0017	0.8388 ± 0.0056	0.8018 ± 0.0034	0.8199 ± 0.0028
BERT-Classification	0.00003	0.1	N/A	0.8547 ± 0.0027	0.9072 ± 0.0037	0.8405 ± 0.0026	0.8726 ± 0.0024

*Note.* LR: learning rate; DR: dropout rate; MC: number of memory cells of RNN; N/A: not applicable.

**Table 4:** Model performance on HFDS test data sets.

Model	Accuracy	Precision	Recall	F1-score
Encoder (Char)-Decoder (Char)	0.8641 ± 0.0065^a,b^	0.8656 ± 0.0084^a,b^	0.8555 ± 0.0062^a,c^	0.8605 ± 0.0056^a,b^
Encoder (Char)-Decoder (Label)	0.8558 ± 0.0070^a,b^	0.8727 ± 0.0038^a,b^	0.8463 ± 0.0062^a,b^	0.8593 ± 0.0043^a,b^
Encoder (Word)-Decoder (Label)	0.8487 ± 0.0046^a,b^	0.8678 ± 0.0076^a,b^	0.8377 ± 0.0054^a,b^	0.8525 ± 0.0059^a,b^
Encoder (Word)-Decoder (Word)	0.8451 ± 0.0035^a,b^	0.8472 ± 0.0056^a,b^	0.8345 ± 0.0036^a,b^	0.8408 ± 0.0023^a,b^
Encoder (Char)-Classification	0.8311 ± 0.0078^c^	0.8937 ± 0.0072^c^	0.8342 ± 0.0077^c^	0.8629 ± 0.0045^c^
Encoder (Word)-Classification	0.8294 ± 0.0079^c^	0.8983 ± 0.0055^c^	0.8302 ± 0.0070^c^	0.8629 ± 0.0038^c^
BERT-UniLM (Char)	0.8914 ± 0.0059^c^	0.8983 ± 0.0042^c^	0.8855 ± 0.0077^c^	0.8918 ± 0.0043^c^
BERT-UniLM (Label)	0.8829 ± 0.0046^c^	0.8909 ± 0.0069^c^	0.8773 ± 0.0044^c^	0.8840 ± 0.0036^c^
**BERT-Classification**	**0.9051** **±** **0.0039**	**0.9118** **±** **0.0033**	**0.9028** **±** **0.0046**	**0.9073** **±** **0.0033**

*Note.* The results are expressed as mean ± SD, and the threshold value of the sigmoid function was 0.2. ^a^*P* < 0.05, compared with BERT-UniLM (Char); ^b^: *P* < 0.05, compared with BERT-UniLM (Label). ^c^*P* < 0.05, compared with BERT-Classification.

**Table 5:** Model performance on TDS test data sets.

Model	Accuracy	Precision	Recall	F1-score
Encoder (Char)-Decoder (Char)	0.7980 ± 0.0078^a,b^	0.7876 ± 0.0147^a,b^	0.7678 ± 0.0088^a,b^	0.7775 ± 0.0106^a,b^
Encoder (Char)-Decoder (Label)	0.7974 ± 0.0050^a,b^	0.8060 ± 0.0081^a,b^	0.7690 ± 0.0062^a,b^	0.7870 ± 0.0056^a,b^
Encoder (Word)-Decoder (Label)	0.7892 ± 0.0069^a,b^	0.7979 ± 0.0100^a,b^	0.7595 ± 0.0066^a,b^	0.7782 ± 0.0074^a,b^
Encoder (Word)-Decoder (Word)	0.7904 ± 0.0079^a,b^	0.7805 ± 0.0122^a,b^	0.7594 ± 0.0078^a,b^	0.7698 ± 0.0092^a,b^
Encoder (Char)-Classification	0.7559 ± 0.0056^c^	0.8560 ± 0.0125^c^	0.7278 ± 0.0057^c^	0.7866 ± 0.0058^c^
Encoder (Word)-Classification	0.7652 ± 0.0042^c^	0.8557 ± 0.0065^c^	0.7364 ± 0.0038^c^	0.7915 ± 0.0028^c^
BERT-UniLM (Char)	0.8274 ± 0.0087^c^	0.8152 ± 0.0115^c^	0.8043 ± 0.0082^c^	0.8097 ± 0.0094^c^
BERT-UniLM (Label)	0.8248 ± 0.0045^c^	0.8230 ± 0.0066^c^	0.7970 ± 0.0056^c^	0.8098 ± 0.0037^c^
**BERT-Classification**	**0.8568** **±** **0.0029**	**0.8870** **±** **0.0039**	**0.8298** **±** **0.0037**	**0.8574** **±** **0.0026**

*Note.* The results are expressed as mean ± SD, and the threshold value of the sigmoid function was 0.1. ^a^*P* < 0.05, compared with BERT-UniLM (Char); ^b^*P* < 0.05, compared with BERT-UniLM (Label). ^c^*P* < 0.05, compared with BERT-Classification.

**Table 6:** The top 5 and 10 recall on the test set.

Model	HFDS		TDS	
	Top 5 recall	Top 10 recall	Top 5 recall	Top 10 recall
Encoder (Char)-Classification	0.9692 ± 0.0028^*∗*^	0.9818 ± 0.0013^*∗*^	0.8906 ± 0.0045^*∗*^	0.9164 ± 0.0037^*∗*^
Encoder (Word)-Classification	0.9635 ± 0.0025^*∗*^	0.9785 ± 0.0015^*∗*^	0.8928 ± 0.0046^*∗*^	0.9195 ± 0.0038^*∗*^
**BERT-Classification**	**0.9758** **±** **0.0012**	**0.9858** **±** **0.0011**	**0.9212** **±** **0.0019**	**0.9426** **±** **0.0015**

*Note.* The results are expressed as mean ± SD. ^*∗*^*P* < 0.05, compared with BERT-Classification.

**Table 7:** Comparison of the BERT-Classification model with other models.

Model	Accuracy	Precision	Recall	F1-score
Jaccard similarity	0.49188	0.65251	0.54722	0.54317
Word2Vec with cosine	0.6424 ± 0.0019^*∗*^	0.7365 ± 0.0093^*∗*^	0.6906 ± 0.0036^*∗*^	0.6724 ± 0.0047^*∗*^
DNorm	0.8572 ± 0.0050^*∗*^	0.8694 ± 0.0087^*∗*^	0.8602 ± 0.0072^*∗*^	0.8555 ± 0.0061^*∗*^
Transition-based model	0.7980 ± 0.0056^*∗*^	0.8256 ± 0.0081^*∗*^	0.7970 ± 0.0051^*∗*^	0.7937 ± 0.0050^*∗*^
RNN-CNNs-CRF	0.8852 ± 0.0036^*∗*^	0.8755 ± 0.0035^*∗*^	0.8724 ± 0.0032^*∗*^	0.8645 ± 0.0034^*∗*^
BERT-based ranking	0.9264 ± 0.0057	0.9413 ± 0.0056	0.9321 ± 0.0072^*∗*^	0.9313 ± 0.0065^*∗*^
**BERT-Classification**	**0.9300** **±** **0.0019**	**0.9473** **±** **0.0023**	**0.9380** **±** **0.0021**	**0.9378** **±** **0.0021**

*Note.* The test results are expressed as mean ± SD. Each model was repeated 10 times, except for Jaccard similarity. ^*∗*^*P* < 0.05, compared with BERT-Classification.

**Table 8:** The top 5 and 10 recall of models.

Model	Top 5 recall	Top 10 recall
Jaccard similarity	0.57312	60857
Word2Vec with cosine	0.9145 ± 0.0025^*∗*^	0.9524 ± 0.0035^*∗*^
DNorm	0.9702 ± 0.0006^*∗*^	0.9858 ± 0.0008^*∗*^
BERT-based ranking	0.9852 ± 0.0049	0.9910 ± 0.0050
**BERT-Classification**	**0.9864** **±** **0.0044**	**0.9921** **±** **0.0048**

*Note.* The test results are expressed as mean ± SD. Each model was repeated 10 times, except for Jaccard similarity. ^*∗*^*P* < 0.05, compared with BERT-Classification.

**Table 9:** Model comparison.

Model	Modeling concept	Complex symptoms ^*a*^	Retrieval ^*b*^	Overall performance ^*c*^
Jaccard similarity	Similarity matching	×	√(0.61)	×
Word2vec with cosine	Similarity matching	×	√(0.95)	×
Encoder-Classification (our)	Text classification	√(0.89)	√(0.98)	√(0.86)
Encoder-Decoder (our)	Sequence generation	√(0.87)	×	√(0.86)
DNorm	Similarity matching	×	√(0.99)	×
Transition-based model	NER	×	×	×
Bi-LSTM-CNNs-CRF	NER	×	×	×
BERT-based ranking	Similarity matching	×	√(0.99)	×
BERT-UniLM (our)	Sequence generation	√(0.90)	×	√(0.89)
**BERT-Classification** (our)	**Text classification**	**√(0.92)**	**√(0.99)**	**√(0.91)**

*Note.* ^*a*^ means the ability to handle complex symptoms, if the model has this ability, it is evaluated for performance using F1-score; ^*b*^ means the ability to retrieve normalized symptoms, if the model has this ability, it is evaluated for performance using top 10 recall; ^*c*^ stands for the overall performance of normalizing single symptoms and complex symptoms, if the model has the ability of handling single symptoms and complex symptoms, it is evaluated by F1-score. √ indicates that the model has this ability or can be evaluated for overall performance. × indicates that the model does not have this ability or cannot be evaluated for overall performance.

## Data Availability

All the data and materials used in the current study are available from the corresponding author on reasonable request.

## Abbreviations

BERT: Bidirectional encoder representation from transformers
Bi-LSTM: Bidirectional long short-term memory
DR: Dropout rate
FN: False negative
FP: False positive
HFDS: High-frequency data sets
LR: Learning rate
MC: Memory cell
N/A: Not applicable
NLP: Natural language processing
RNN: Recurrent neural network
SD: Standard deviation
TCM: Traditional Chinese medicine
TDS: Total data sets
TP: True positive
UniLM: Unified language model.

## References

[1] Wang Y., Yu Z., Jiang Y., Xu K., Chen X. (2010). Automatic symptom name normalization in clinical records of traditional Chinese medicine. *BMC Bioinformatics*.

[2] Chu X., Sun B., Huang Q., Peng S., Zhou Y., Zhang Y. (2020). Quantitative knowledge presentation models of traditional Chinese medicine (TCM): a review. *Artificial Intelligence in Medicine*.

[3] Xiao X.-X., Yan J.-F., Liu D.-B. (2018). Abstraction of data elements of clinical symptoms in Chinese medicine. *Digital Chinese Medicine*.

[4] National Administration of Traditional Chinese Medicine (2020). *National Administration of Traditional Chinese Medicine National Health Commission on Printing and Distributing the Classification and Code of traditional Chinese medical Disease and pattern and Clinic Terminology of Traditional Chinese Medical Diagnosis and Treatment*.

[5] Vathsala M. K., Holi G. (2020). RNN based machine translation and transliteration for twitter data. *International Journal of Speech Technology*.

[6] Cai L.-Q., Wei M., Zhou S.-T., Yan X. (2020). Intelligent question answering in restricted domains using deep learning and question pair matching. *IEEE Access*.

[7] Arji G., Safdari R., Rezaeizadeh H., Abbassian A., Mokhtaran M., Hossein Ayati M. (2019). A systematic literature review and classification of knowledge discovery in traditional medicine. *Computer Methods and Programs in Biomedicine*.

[8] Sheikhalishahi S., Miotto R., Dudley J. T., Lavelli A., Rinaldi F., Osmani V. (2019). natural language processing of clinical notes on chronic diseases: systematic review. *JMIR Medical Informatics*.

[9] Zhang H., Ni W., Li J., Zhang J. (2020). Artificial intelligence-based traditional Chinese medicine assistive diagnostic system: validation study. *JMIR Medical Informatics*.

[10] Névéol A., Dalianis H., Velupillai S., Savova G., Zweigenbaum P. (2018). Clinical natural language processing in languages other than english: opportunities and challenges. *Journal of Biomedical Semantics*.

[11] Cho H., Choi W., Lee H. (2018). A method for named entity normalization in biomedical articles: application to diseases and plants. *BMC Bioinformatics*.

[12] Park S., Kim D.-Y (2017). Assessing language discrepancies between travelers and online travel recommendation systems: application of the Jaccard distance score to web data mining. *Technological Forecasting and Social Change*.

[13] Leaman R., Islamaj Dogan R., Lu Z. (2013). DNorm: disease name normalization with pairwise learning to rank. *Bioinformatics*.

[14] Wang Q., Ji Z., Wang J. (2020). A study of entity-linking methods for normalizing Chinese diagnosis and procedure terms to ICD codes. *Journal of Biomedical Informatics*.

[15] Lou Y., Zhang Y., Qian T., Li F., Xiong S., Ji D. (2017). A transition-based joint model for disease named entity recognition and normalization. *Bioinformatics*.

[16] Zhao S., Liu T., Zhao S., Wang F. (2019). A neural multi-task learning framework to jointly model medical named entity recognition and normalization. *Proceedings of the AAAI Conference on Artificial Intelligence*.

[17] Chen C.-W., Tseng S.-P., Kuan T.-W., Wang J.-F. (2020). Outpatient text classification using attention-based bidirectional LSTM for robot-assisted servicing in hospital. *Information*.

[18] Dong L., Yang N., Wang W. (2019). Unified language model pre-training for natural language understanding and generation. https://arxiv.org/abs/1905.03197.

[19] Zhang R., Xie Q., Li K. (2016). Design and application of the management platform of the “heritage program of Chinese well-known experts” of China academy of Chinese medical sciences. *World Science and Technology/Modernization of Traditional Chinese Medicine and Materia Medica*.

[20] Pao M. L. (1978). Automatic text analysis based on transition phenomena of word occurrences. *Journal of the American Society for Information Science & Technology*.

[21] Sutskever I., Vinyals O., Le Q. V. Sequence to sequence learning with neural networks.

[22] Luong M.-T., Pham H., Manning C. D. Effective approaches to attention-based neural machine translation.

[23] Liu S., Zhou L., Li C. (2021). Research on modeling of traditional Chinese medicine word segmentation model based on sentence piece. *World Journal of Traditional Chinese Medicine*.

[24] Devlin J., Chang M. W., Lee K., Toutanova K. (2019). BERT: pre-training of deep bidirectional transformers for language understanding. https://arxiv.org/abs/1810.04805.

[25] Kingma D., Ba J. Adam: a method for stochastic optimization.

